# Somatostatin Receptor Scintigraphy in a Patient with Myocarditis

**DOI:** 10.4274/mirt.galenos.2019.03164

**Published:** 2021-02-09

**Authors:** Abdullatif Amini, Firoozeh Dehdar, Esmail Jafari, Ali Gholamrezanezhad, Majid Assadi

**Affiliations:** 1 Bushehr Medical Heart Center, Bushehr University of Medical Sciences, Department of Cardiology, Bushehr, Iran; 2 Bushehr University of Medical Sciences, Bushehr Medical University Hospital, The Persian Gulf Nuclear Medicine Research Center, Department of Molecular Imaging and Radionuclide Therapy (MIRT), Bushehr, Iran; 3 University of Southern California, Keck School of Medicine, Department of Diagnostic Radiology, Los Angeles, USA

**Keywords:** Myocarditis, somatostatin receptor scintigraphy, Tc-99m octreotide, myocarditis, SPECT

## Abstract

We report a case of myocarditis imaged with technetium-99m octreotide cardiac single-photon emission computed tomography which showed diffuse uptake in the myocardium, indicating inflammatory reaction to myocardial damage. Somatostatin receptor scintigraphy of the heart could be considered in patients with suspected cardiac inflammation. This could facilitate early diagnosis and guide appropriate treatment.

## Abstract (tr)

Bu çalışmada, miyokardiyal hasara enflamatuvar reaksiyonu gösteren, miyokardda yaygın alım gösteren teknesyum-99m oktreotid kardiyak tek foton emisyonlu bilgisayarlı tomografi ile görüntülenen bir miyokardit olgusunu sunuyoruz. Miyokardiyal enflamasyondan şüphelenilen hastalarda göğüs ağrısı değerlendirilirken kalbin somatostatin reseptör sintigrafisi düşünülebilir. Bu, erken teşhisi kolaylaştırabilir ve uygun tedaviyi sağlayabilir.

## Figures and Tables

**Figure 1:**
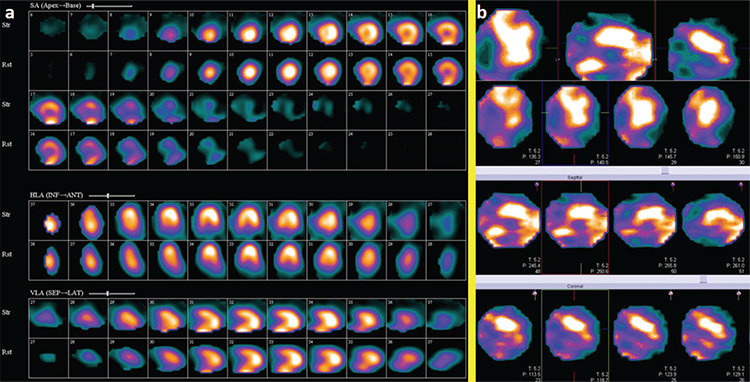
A 21-year-old male patient was admitted in our hospital for mild chest discomfort since 1 month ago and a 2-mm ST-segment elevation in all leads, except in AVR and V1. His workup was performed elsewhere. At that time, he experienced severe acute chest pain accompanied by dyspnea. The patient was afebrile. He had no history of a viral or other infection and drug abuse. On history assessment, he had tachycardia, and his blood pressure was in the normal range. Cardiac laboratory tests, especially troponin and creatine kinase, were markedly elevated, while an echocardiographic examination was unremarkable with an ejection fraction of 55%. The patient has received high-dose antiinflammatory drugs, which was probably associated with acute pericarditis. The aforementioned cardiac biomarkers decreased gradually within a few days of admission, and the patient was discharged that time. On recent admission, myocardial perfusion imaging using technetium-99m (Tc-99m) methoxyisobutylisonitrile (MIBI) single photon emission computed tomography (SPECT) was carried out, and the results were unremarkable, but revealed ejection fraction of >55%. (a) Due to probable pericarditis/myocarditis, tomographic somatostatin receptor imaging with Tc-99m octerotide was performed, which was suggestive of persistent inflammatory reaction. There was diffuse myocardial Tc-99m-octerotide uptake at the utmost anterior region (b), while the pattern in atherosclerosis usually is focal, depending on the involved artery. On follow-up visits, the symptoms completely improved, and all laboratory tests became normal. Myocarditis is characterized with myocardial inflammation without ischemia or infarction, and several causes have been identified, with viral infections being the most frequent (1). Currently, endomyocardial biopsy (EMB) is the gold standard for distinguishing myocarditis; nevertheless, it has very low sensitivity of only 20%-30% with a noticeable procedure-related risk (2). Cardiac magnetic resonance imaging is the standard imaging technique in revealing myocarditis, and it can detect several characteristics of myocarditis; nonetheless, it has some main drawbacks, especially in the detection of chronic myocarditis with accuracies as low as 50%. Furthermore, it cannot show the inflammatory activity, which is highly necessary for monitoring of therapeutic responses (2). ^18^Fluorine-fluorodeoxyglucose (^18^F-FDG)-positron emission tomography (PET)/CT can show acute myocardial inflammation suggestive of active myocarditis. In addition, based on the low yield of random EMB, PET-guided myocardial biopsy may be another indication for ^18^F-FDG-PET/CT in myocarditis. Currently, the research on hybrid PET/magnetic resonance imaging for diagnosing myocarditis is an active area of research (3). Moreover, due to the low specificity of ^18^F-FDG-PET, novel PET tracers such as (11C)-methionine for imaging of myocarditis are being explored (4). Similarly, targeting of somatostatin receptor 2 has shown promising findings in a clinical pilot investigation (5). With regard to radionuclide imaging, gallium-67-citrate and 111In-antimyosin scintigraphy have demonstrated some efficacy in the diagnosis of myocardial inflammation and necrosis, respectively, but their application in the evaluation of myocarditis has deceased largely due to limited specificity and availability (1). In addition, very few cases on somatostatin receptor scintigraphy (SRS) in myocarditis are reported in the literature (1). The most notable explanation underlying these processes could be related to the expression of somatostatin receptor subtype 2 on activated lymphocytes and macrophages, an abundant cell type in the atherosclerotic plaque and myocarditis; with this difference, vulnerable atherosclerotic plaque is usually a focal process, while myocarditis is a diffuse process (6,7). In the field of nuclear imaging, ^18^F-FDG-PET/CT and leukocyte scintigraphy are the most commonly applied techniques in these situations. Other innovative modalities such as bacteria-specific imaging agents and C-X-C motif chemokine receptor CXCR4 have shown promising results in trial studies (8). Briefly, cardiac SRS may be a valuable imaging modality in the assessment of myocarditis, especially when other standard imaging techniques are unavailable or unsuitable.

**Figure 2:**
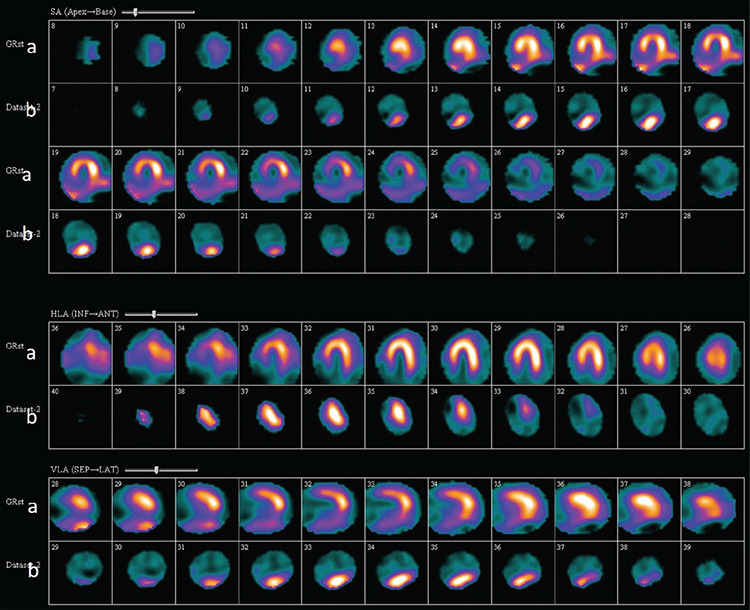
(a) Myocardial perfusion imaging using Tc-99m-MIBI SPECT at rest in a 51-year-old man. Short, vertical, and horizontal slices in the top rows, showing severely decreased uptake in the inferior and inferoseptal walls. (b) Myocardial SSTR imaging with Tc-99m labeled octreotide SPECT in the same patient. Short axis, vertical, and horizontal slices in the lower rows showed uptake in the inferior region suggesting vulnerable plaque.

## References

[1] Moralidis E, Mantziari L, Gerasimou G, Styliadis IH, Gotzamani-Psarrakou A (2012). Somatostatin analogue scintigraphy in a patient with viral myocarditis. Hell J Nucl Med.

[2] Lurz P, Eitel I, Adam J, Steiner J, Grothoff M, Desch S, Fuernau G, de Waha S, Sareban M, Luecke C, Klingel K, Kandolf R, Schuler G, Gutberlet M, Thiele H (2012). Diagnostic performance of CMR imaging compared with EMB in patients with suspected myocarditis. JACC Cardiovasc Imaging.

[3] Nensa F, Poeppel TD, Krings P, Schlosser T (2014). Multiparametric assessment of myocarditis using simultaneous positron emission tomography/magnetic resonance imaging. Eur Heart J.

[4] Maya Y, Werner RA, Schütz C, Wakabayashi H, Samnick S, Lapa C, Zechmeister C, Jahns R, Jahns V, Higuchi T (2016). 11C-Methionine PET of Myocardial Inflammation in a Rat Model of Experimental Autoimmune Myocarditis. J Nucl Med.

[5] Lapa C, Reiter T, Li X, Werner RA, Samnick S, Jahns R, Buck AK, Ertl G, Bauer WR (2015). Imaging of myocardial inflammation with somatostatin receptor based PET/CT - A comparison to cardiac. MRI Int J Cardiol.

[6] Malmberg C, Ripa RS, Johnbeck CB, Knigge U, Langer SW, Mortensen J, Oturai P, Loft A, Hag AM, Kjær A (2015). 64Cu-DOTATATE for Noninvasive Assessment of Atherosclerosis in Large Arteries and Its Correlation with Risk Factors: Head-to-Head Comparison with 68Ga-DOTATOC in 60 Patients. J Nucl Med.

[7] Spiridonidis T, Patsouras N, Papandrianos N, Symeonidis A, Apostolopoulos DJ (2008). Tc-99m Depreotide SPECT/CT depicts myocardial involvement in a case of thrombotic thrombocytopenic purpura. Clin Nucl Med.

[8] Kircher M, Lapa C (2017). Novel Noninvasive Nuclear Medicine Imaging Techniques for Cardiac Inflammation. Curr Cardiovasc Imaging Rep.

